# Comparison of Malnutrition Indicators and Associated Socio-Demographic Factors among Children in Rural and Urban Public Primary Schools in South Africa

**DOI:** 10.3390/children10111749

**Published:** 2023-10-27

**Authors:** Mosebudi Olga Hlahla, Lindy Agatha Kunene, Peter Modupi Mphekgwana, Sphiwe Madiba, Kotsedi Dan Monyeki, Perpetua Modjadji

**Affiliations:** 1 Department of Public Health, School of Health Care Sciences, Sefako Makgatho Health Sciences University, 1 Molotlegi Street, Ga-Rankuwa, Pretoria 0208, South Africa; 2 Research Administration and Development, University of Limpopo, Polokwane 0700, South Africa; 3 Faculty of Health Sciences, University of Limpopo, Polokwane 0700, South Africa; 4 Department of Physiology and Environmental Health, University of Limpopo, Polokwane 0700, South Africa; 5 Non-Communicable Disease Research Unit, South African Medical Research Council, Cape Town 7505, South Africa; 6 Department of Life and Consumer Sciences, College of Agriculture and Environmental Sciences, University of South Africa, Roodepoort, Johannesburg 1709, South Africa

**Keywords:** malnutrition, socio-demographic factors, primary school children, rural–urban settings, South Africa

## Abstract

Most children in South Africa attending public schools are predisposed to malnutrition due to poor infrastructure and social inequality. This is despite the implementation of the National School Nutrition Programme to address barriers to learning associated with hunger and malnutrition and the National Development Plan to reduce child malnutrition through provision of social grants. In view of this, we compared malnutrition indicators and associated socio-demographic factors among children in rural Mpumalanga and urban Gauteng in South African public primary schools selected using a multistage cluster random sampling. A validated researcher-administered questionnaire was used to collect socio-demographic data of caregivers, along with primary school children data collected on age, sex, learning grade, and anthropometric measures. Malnutrition indicators, which are stunting (low height-for-age z-scores), underweight (low weight-for-age z-scores), thinness (low body-mass-index-for-age z-scores), and overweight/obesity (high body mass index) were computed using WHO Anthro Plus 1.0.4 and data were analyzed using Stata 18. A total of 903 children (rural = 390 and urban = 513) with a mean age of 10 ± 2 years in the foundation phase (learning grades one to three) and the intermediate learning phase (learning grades four to seven) participated with their caregivers (mean age: 39 ± 8 years). Significant levels of poor socio-demographic status were observed among caregivers living in the rural setting compared to in the urban setting. Overall, thinness (18%), stunting (12%), underweight (10%), and overweight/obesity (24%) were observed among school children. Children in the rural schools had a significantly higher prevalence of stunting (20% vs. 3%; *p* < 0.0001), underweight (17% vs. 2%; *p* < 0.0001) and thinness (28% vs. 7%; *p* < 0.001) than their urban counterparts. In the urban, the odds of stunting, underweight and thinness were less among school children, while overweight/obesity was twice as likely in the urban setting. The multivariate final model showed lower odds of underweight [adjusted odds ratio (AOR) = 0.16; 95% confidence interval (CI): 0.06–0.42] and stunting [AOR = 0.33; 95% CI: 0.13–0.87] in the urban compared to the rural schools. The association of stunting with sex [AOR =0.53; 95% CI: 0.30–0.94] and the intermediate learning phase [AOR = 7.87; 95% CI: 4.48–13.82] was observed in the rural setting, while thinness was associated with living in households with an income of USD 52.51 to USD 262.60/month [AOR = 2.89; 95% CI: 1.01–8.24] and receiving the child social grant [AOR = 2.49; 0.90–6.86] in the urban setting. Overweight/obesity was associated with living in a household with an income of USD 52.51 to USD 262.60/month [AOR = 1.80; 95% CI: 1.02–3.10]. The findings suggest nutritional intervention approaches that are accustomed to the context of settings to effectively tackle malnutrition.

## 1. Introduction

Low-and middle-income countries (LMICs) continue to experience undernutrition co-occurring with overnutrition, called the double burden of malnutrition [1,2,3,4]. While undernutrition indicators include stunting (low height-for-age), underweight (low weight-for-age), thinness (low body mass index-for-age) and nutrients deficiencies [5], overnutrition is characterized by overweight/obesity (high body mass index-for-age) and the overconsumption of calories [6,7,8,9]. Both undernutrition and overnutrition (i.e., malnutrition) are multifactorial [10] and lead to impaired metabolism due to the disproportionality between food consumption and energy requirement in an individual [11,12]. In Sub-Saharan Africa (SSA) the prevalence of undernutrition among school-aged children ranges between 7% and 27% [13,14,15,16,17], and overnutrition between 13% and 21% [18,19,20]. Similarly, rates of childhood overweight/obesity (8.6%–54%) are alarming in South Africa [21,22,23,24], including persistent stunting (22%), underweight (27%) and thinness (25%) reported in school-aged children [2,4]. 

Malnutrition remains notable among children from low socio-economic backgrounds, living in the rural setting, and attending public (i.e., government) schools [2,4,25]. In SSA, residence is a health determinant and there is an advantage of children living in the urban setting in association with malnutrition [13,26,27]. A better nutritional status is associated with urban settings than rural settings [28,29]. Nonetheless, differences in the prevalence of undernutrition between urban, rural and commercial farms are evident in South African children [29], and other African countries such as Ethiopia [13,14], Nigeria [15] and Cameroon [30]. In both rural and urban settings, nutritional outcomes have been more associated with socio-economic characteristics in LMICs [31,32,33] but more in the rural in South Africa [34,35]. Evidently, a lower income restricts access to healthy diets and makes available more access to unhealthy high-energy-dense foods [36], while favorable socio-economic conditions positively influence infant feeding and caring practices in favor of the urban setting [28]. 

Furthermore, for almost a decade, the physical school environment has been reported as the primary determinant of children’s nutritional and health status [37]. South Africa has three types of school environments, aside from international schools, which are public schools, governing body-funded schools, and private schools [38]. Children attending public schools in quintiles 1 to 3 (i.e., schools ranked as the poorest or lowest in terms of socio-economic status) receive school meals through the National School Nutrition Programme (NSNP). The NSNP is a government initiative that commenced in 1994 and is designed to address barriers to learning associated with hunger and malnutrition by providing nutritious meals [39,40]. School age is a critical stage for growth spurts among children affected by suboptimal food intake, access, and distribution as well as pathogenic infections, making them vulnerable to malnutrition [41]. Malnutrition predisposes school-aged children to impaired school performance and intellectual achievement, delayed maturation, etc. [42,43,44,45], as well as developing non-communicable diseases (NCDs), even later in life [46]. 

In terms of NSNP efforts, it seems the program is clouded by challenges regarding food quantity and quality, irregular food supply, a lack of diet variety, the bureaucratic system, a lack of uniform procurement issues in provinces, and corruption and theft [39,47,48,49]. At the same time, the school food and nutrition environment in South Africa is still not conducive for promoting healthy eating and meeting nutritional requirements, exacerbating malnutrition as a public health challenge in school children [10,50]. Despite these known facts, studies are still minimal on the magnitude, determinants, and context of malnutrition among school children [2,4,40], who are often excluded from health and nutrition surveillance. Therefore, continuous assessment of malnutrition among school children can never be overemphasized. In view of potential contextual differences of malnutrition indicators between public schools in rural and urban settings, this study aimed to compare malnutrition indicators and the associated socio-demographic factors among children in rural Mpumalanga and urban Gauteng in South African public primary schools. This study serves as one of the very few baseline studies in South Africa indicating potential setting differences to suggest contextual intervention programs when addressing malnutrition, in line with the United Nations’ (UN) sustainable development goal two (SDG-2) [51]. 

## 2. Methods

### 2.1. Study Design, Population, and Settings

We conducted a cross-sectional survey starting with the Ekurhuleni sub-district in the Gauteng urban (GUS) setting in 2017, and subsequently expanded to the Thaba-Chweu sub-district in the Ehlanzeni district, in the Mpumalanga rural (MRS) setting in 2018. Mpumalanga is the second-smallest province after Gauteng, with a population of approximately 4 million, and has three districts, namely Ehlanzeni, Nkangala and Gert Sibande [52]. Gauteng is one of the nine provinces in South Africa but comprises the largest portion of the South African population, estimated at 13 million [53]. The proximity of the two provinces with evidence of poor nutritional outcomes reported among children in general in neighboring districts, and the differences in the context of provinces, warranted a comparative analysis of malnutrition indicators and socio-demographic-associated factors among school-aged children. Also, we considered that most if not all public primary schools in the rural and urban settings belong to quintile three, meaning no school fee is charged while receiving financial assistance from the South African government [49]. 

The above projects were an extension of a doctoral project on “Growth patterns among primary school children in the Limpopo province, South Africa” (Growth patterns project) approved by the Sefako Makgatho Health Sciences University Research and Ethics Committee, on 4 August 2016 (SMUREC/H/161/2016: PG), active between 2016 and 2022. Among its specific objectives, the Growth patterns project aimed to best understand the magnitude of malnutrition through multilevel influences among school-aged children paired with their caregivers using a convergent parallel mixed-methods design, detailed in Modjadji and Madiba. Furthermore, it developed a framework to study malnutrition using a convergent parallel mixed-methods approach [10]. First the quantitative survey determined the magnitude of malnutrition and associated factors among school children paired with their biological mothers (*n* = 508 child–mother pairs), using a multistage sampling technique. Parallel to the quantitative survey, a qualitative study explored the mothers’ insights into child growth and nutrition in seven FGDs (*n* = 54) consisting of mothers who were selected based on their child being malnourished. The quantitative results and qualitative findings were analyzed independently, and thereafter, mixed-methods integration was achieved through the convergence of six quantitative constructs with ten qualitative themes using a joint display analysis to compare the findings and generate meta-inferences [54]. The methodological aspects of this current investigation were adopted from the above-mentioned doctoral project published in several papers [2,4,10,55,56] in terms of data collection tools and processes. In a nutshell, the concept was informed by the UNICEF conceptual framework for malnutrition [57] and Bronfenbrenner’s social ecological model for child growth and development [58] influenced by the school, and household environments because of the diversity, multidimensional, and interrelated causes of childhood malnutrition [59].

### 2.2. Sample Size and Sampling Technique

We used a validated Rao soft sample size calculator suitable for calculation of sample sizes in surveys [60] to estimate a minimum representative sample size for school children. Considering the combined population size of approximately 70,000 from the Education Management Information System for both Gauteng and Mpumalanga 2017/2018, and a 95% confidence level, a minimum representative sample size of 383 was calculated to achieve the objective of this study, and a final sample of 903 school children was obtained: MRS = 390 and GUS = 513. Primary schools were stratified by the size of enrolment, then by one class per grade, and all school children from grades one to seven in the selected class (i.e., multistage cluster random sampling). This study excluded children who were not of the foundation phase age, had a physical disability that would compromise their stature, and reported to have ill-heath by the caregivers. 

Following ethics approval from the Sefako Makgatho Health Sciences University Research and Ethics Committee (SMUREC) and permission from the provincial department of education in Mpumalanga and Gauteng, we visited the selected primary schools to seek approval from the principals and school governing bodies. Thereafter, through schools’ systems, school children were recruited at their respective schools with the help of nutrition teachers liaising with class teachers to distribute consent forms and information leaflets to the caregivers to seek written consent for participation with their children through schools’ systems. From the returned and signed consent forms by the caregivers, we further selected school children randomly in the selected classes. School children were further engaged on the procedure of this study and preparations for data collection in local languages, and those who agreed to participate in this study gave assent accompanied by their caregivers’ consent. All processes were performed during breaks and after schools, without interrupting learning.

### 2.3. Data Collection and Tools

Data on malnutrition indicators, socio-demographic status, household and child information were collected from the caregivers using a developed structured interviewer-administered questionnaire [57,61,62], translated to siSwati and isZulu—common languages in the settings—by a translator conversant with the two languages and English. After ensuring content, construct, and face validity through engaging nutrition experts, pilot studies were conducted in both settings to pretest a questionnaire while training the four research assistants on conducting interviews and measuring the anthropometry of caregivers and school children in the two smallest primary schools that did not form part of the main study. Results from the pilot study informed the main study after implementing a few changes in the questionaries. Using standard procedures [63], three measurements for weights and heights were taken at once from school children and their caregivers, and the averages were recorded. Body weight was measured to the nearest 0.1 kg using a calibrated DQUIP Smart Scale Dual Display Silver 427 (manufacturer: Dis-Chem Pharmacies, South Africa) while the participant was standing in the center of the scale, bare footed and with minimal clothing. Height was measured to the nearest 0.1 cm using a height measuring board (i.e., Seca 216 stadiometer; manufacturer: Baseline, South Africa) while a participant was standing with heels, buttocks, and upper back against the measuring board, and shoulders relaxed, legs straight, arms at sides, and looking straight ahead to achieve a Frankfort plane. Waist circumference was measured to the nearest 0.1 cm while the participant was wearing thin clothing around the abdomen and hips, and the tape measure was placed between the top of the hipbone and the bottom of the ribs. Hip circumference was measured to the nearest 0.1 cm while the participant was standing straight with legs together, and the tape measure was placed around the widest part of the hip (above the gluteal fold), and straight around the back [63]. 

Malnutrition indicators of children were assessed using age, sex, and anthropometric measurements and computed height-for-age z-scores (HAZ; stunting), weight-for-age z-scores (WAZ; underweight), and body-mass-index-for-age z-scores (BMI; thinness, overweight and obesity) using WHO Anthro Plus 1.0.4, and analyzed according to WHO Child Growth Standards and the WHO Reference [64]. Stunting, underweight and thinness were considered as z-scores less than or equal to −2SD, while overweight and obesity were taken as z-scores of +2SD and + 3SD, respectively [64]. For caregivers, overweight was defined as BMI of 25 to 29.9 kg/m^2^, and obesity as BMI ≥ 30 kg/m^2^, and underweight as BMI < 18.5 kg/m^2^, calculated as weight in kilograms divided by height in meters squared [65]. Abdominal obesity was estimated through a waist circumference ≥ 88 cm, waist–hip ratio (WHR) ≥ 0.85, and waist-to-height ratio (WHtR) < 0.5 [66,67]. All data collection processes were performed after school during the week (i.e., Monday to Friday), and during weekends (i.e., Saturday and Sunday) to cater for caregivers who were not available during the week. After completing data collection, fruits (banana or apple or pear) were given to a caregiver and a child. 

### 2.4. Statistical Analysis

STATA 18 (StataCorp. 2023. Stata Statistical Software: Release 18. College Station, TX, USA) was used to analyze data. The median HAZ, WAZ and BAZ scores of children were plotted by learning grades to assess malnutrition indicators as they advance in learning, following identifying missing data (using complete case analysis) and assessing distributions of variables (using Shapiro Francia). Bivariate (Chi square and Fisher’s exact (for cases lower than five in a cell) tests) and backward stepwise multivariable logistic regression were used to assess the association of malnutrition indicators with socio-demographic factors in rural and urban schools. The *p*-value was statistically significant at less than 0.05. Results are presented as frequencies (*n*) and percentages (%). 

### 2.5. Ethics Statement

This study adhered to the principles of the Declaration of Helsinki for human subjects’ research [68]. For the GUS, ethics approval was obtained in 2017 (SMUREC/H/118/2017: PG) and subsequently obtained in 2018 for MRS (SMUREC/H/116/2018: PG) both from the Sefako Makgatho Health Sciences University Research and Ethics Committee (SMUREC). Further permissions were obtained from the departments of education for the Mpumalanga and Gauteng provinces, as well as from the principals of the schools’ and the governing bodies. 

## 3. Results

### 3.1. Characteristics of Caregivers and School Children

A total of 903 school children [MRS; rural =390 and GUS; urban = 513] made up of 441 boys and 462 girls participated in this study with their caregivers. The mean age of caregivers was 39 ± 8 years, and socio-demographic status was significantly better in the urban setting in terms of caregivers’ marital status (48% vs. 22%; *p* < 0.0001), tertiary education level (46% vs. 21%; *p* < 0.0001), employment status (65% vs. 34%; *p* < 0.0001), and living in a household with an income of USD 262.60–USD 525.20/month (37% vs. 6%; *p* < 0.0001) compared to in the rural setting, respectively. Half of the children were receiving the child social grant (CSG), which was more common among rural children compared to among urban children (77% vs. 23; *p* < 0.0001). Results are presented as the frequency (percentage); *n* (%), with *p*-values (Table 1). 

In Table 2, characteristics and malnutrition indicators of children are compared by setting. Children were distributed across learning grades one to three (i.e., foundational phase, *n* = 526), and learning grades four to seven (i.e., intermediate phase (*n* = 377). Their overall mean age was 10 ± 2 years (rural: 9 ± 2 years and urban: 10 ± 2 years) ranging from 6 to 13 years. School children were divided into two age groups of 6–9 (i.e., younger children) and 10–13 years (i.e., older children). Overall, thinness (18%), stunting (12%), and underweight (10%) were observed among school children, while overweight/obesity was 24%. The prevalence of stunting (20% vs. 3%; *p* < 0.0001), underweight (17% vs. 2%; *p* < 0.0001) and thinness (28% vs. 7%; *p* < 0.001) was significantly higher in the rural setting compared to in the urban setting, while almost double the prevalence of overweight/obesity in the rural setting (19%) was observed in the urban setting (28%), *p* < 0.0001. 

### 3.2. Median Values of Malnutrition Indicators

**Figure 1:**
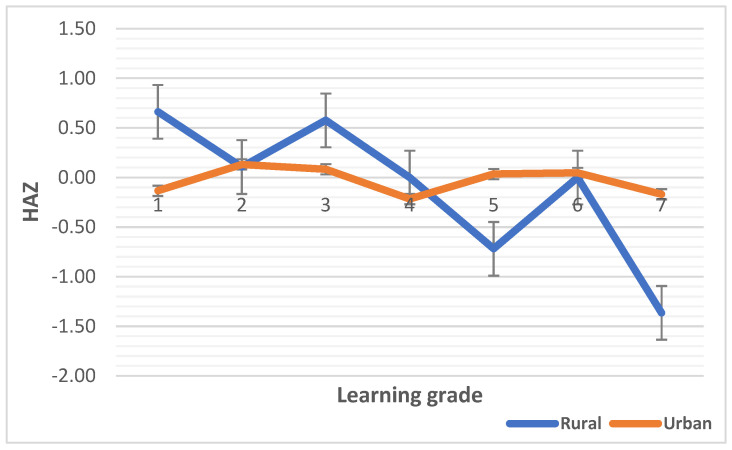
Median HAZ scores by learning grade of rural and urban children. The numbers 1 to 7 are the learning grades of learners in schools.

**Figure 2:**
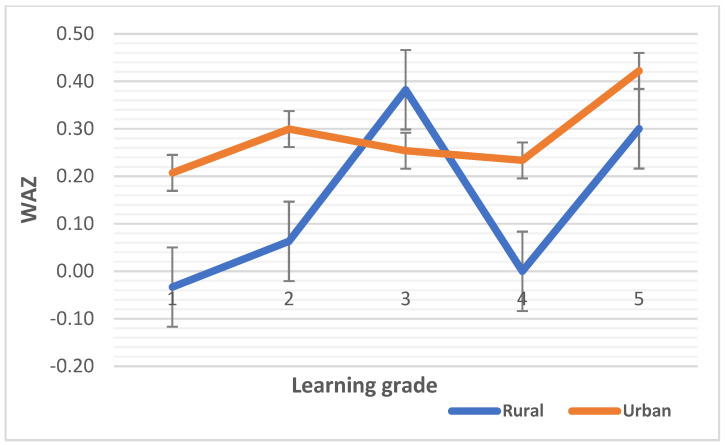
Medians WAZ score by learning grade of rural and urban children. The numbers 1 to 5 are the learning grades of learners in schools.

**Figure 3:**
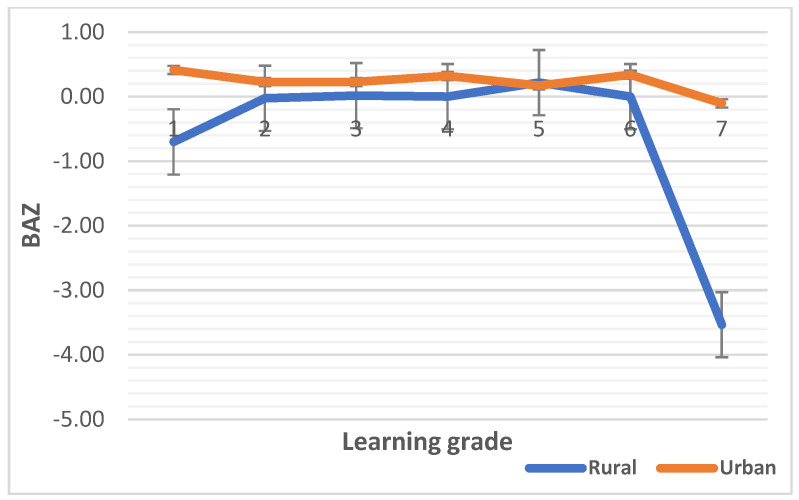
Median BAZ scores by learning grade of rural and urban children. The numbers 1 to 7 are the learning grades of learners in schools.

The median HAZ, WAZ, and BAZ scores are plotted against learning grades in Figure 1, Figure 2 and Figure 3, respectively. The median HAZ score was constantly in the normal range for the urban group but decreased sharply in grades six and seven (intermediate phase) in the rural group (*p* < 0.0001). The median WAZ score of the urban children was higher than that for the rural children and reached a peak in grade three and grade five in the rural setting although not significant (*p =* 0.277). WHO Anthro Plus 1.0.4 software has no reference data for WAZ scores beyond 10 years of age due to the WAZ score not being able to distinguish between height and body mass during this age period [64]. Hence, the median WAZ scores of both rural and urban children are provided for up to grade five. Most children in grades six and seven were beyond 10 years of age in this study. The median BAZ score was constantly higher in urban children and decreased sharply between grades six and seven (*p* < 0.0001). 

### 3.3. The Association of Malnutrition Indicators with Covariates among School Children

#### 3.3.1. Bivariate Association Using Chi-Square/Fisher Exact Tests

Table 3 shows the bivariate associations of malnutrition indicators with caregiver’s socio-demographic status for the two settings. Thinness was significantly associated with household income (*p* = 0.034), and most children in the rural setting were from a household with an income of less than USD 52.51 (≈1000 ZAR)/month. Being employed as a caregiver was associated with overweight/obese children (*p* = 0.020), while receiving social grants was associated with thinness (*p* = 0.029) in the urban setting.

#### 3.3.2. The Association of Malnutrition Indicators Using Multivariable Logistic Regression

Although univariate analysis (*p* < 0.30) of the rural–urban setting association with malnutrition indicators showed that the urban setting was less likely to be associated with stunting, underweight and thinness, and overweight/obesity is twice as likely than in the rural setting, a multivariate final model showed that the urban setting is associated with underweight [adjusted odds ratio (AOR) = 0.16; 95% confidence interval (CI): 0.06–0.42] and stunting [AOR = 0.33; 95% CI: 0.13–0.87]. During multivariable analysis (*p* < 0.05), thinness and overweight/obesity—independent variables in the regression model—were highly correlated with each other (i.e., multicollinearity) and were, as a result, omitted from the final model (Table 4).

In the rural setting, univariate logistic regression (*p* ≤ 0.3) showed that stunting was associated with children’s age, sex, learning grade, receiving the CSG as well as caregivers’ education level, household head and household income/month. Underweight was associated with child’s sex, caregivers’ education level and household head, while thinness was associated with child’s age, learning grade, receiving the CSG, caregivers’ employment status, household income, and access to electricity. In multivariable logistic regression (*p* < 0.05), only stunting was associated with boys [adjusted odds ratio (AOR) = 0.53; 95% confidence interval (CI): 0.30–0.94] and school children in the intermediate phase [AOR = 7.87; 95% CI: 4.48–13.82] (Table 5).

In the urban setting, univariate analysis showed that stunting was associated with receiving the CSG, caregivers’ education level, and household head, and thinness was associated with receiving the CSG, caregivers’ employment status, household income and household head. Multivariable analysis showed that thinness was associated with living in a household with an income of USD 52.51 to USD 262.60/month [AOR = 2.89; 95% CI: 1.01–8.24] and receiving the CSG [AOR = 2.49; 0.90–6.86], while overweight/obesity was associated with living in a household with an income of USD 52.51 to USD 262.60 [AOR = 1.80; 95% CI: 1.02–3.10] (Table 6).

## 4. Discussion

In view of the physical school environment and setting reported as the determinant of children’s nutritional outcomes, we compared malnutrition indicators and associated socio-demographic factors among children in rural Mpumalanga and urban Gauteng in South African public primary schools. Two-thirds (64%) of the South African population live in urban settings and one-third (34%) in rural settings, with more people unemployed in rural areas [69], resulting in a higher incidence of poverty and negatively affecting total household income [69,70]. In this study, the socio-demographic status in the urban setting was significantly better compared to in the rural setting in terms of marital status, education level, employment status, household income and infrastructure. Therefore, rural–urban differences, in addition to poverty and inequality (i.e., socio-economic factors), are major social determinants of health [71]. Our observation is consistent with studies that have documented the relationship between rural/urban settings and socio-demographic factors (marital status, education, employment status, income, etc.), health status (NCDs; diabetes, hypertension, stroke, etc.), lifestyle factors (physical activity, overweight, obesity, etc.) and nutrition status (dietary changes, unhealthy diet, undernutrition., etc.) in LMICs, including South Africa [71,72,73,74,75,76]. In fact, the poorer perceived health status and low quality of life among rural dwellers are attributed to a lower socio-economic status, which might affect the affordability of good nutrition to maintain proper feeding practices as well as access to health care [34,35,75]. 

Since the school environment has the greatest influence on the nutritional status of school children because of the time they spend at school, this environment is important for nutrition interventions, infested as it is by tuck-shops selling snacks—energy-dense foods, fat-rich foods, and sweetened foods—promoting poor dietary practices among school children [50]. School children’s diets are mainly based on starches and lack dietary diversity, which is persistent in some rural communities in South Africa due to poverty [77]. Implementation of the NSNP in South Africa has been challenged by poor infrastructure, such as the absence of kitchens in some schools [39,48,78] as well as the absence of storage, preparation, cooking and eating facilities that meet health and safety standards in schools [47,48,78]. The lack of parental involvement in the implementation of the NSNP, working together with educators, is another issue [49]. Parental involvement in school activities is important for the buy-in of the entire school community because it boosts children’s aspirations, academic performance, and the feeling of belonging to the school [49]. For example, food poisoning among children, which has been reported in provinces such as Limpopo in South Africa, clearly suggests that there are no clear strategies to empower parents to feel a sense of ownership over the NSNP working hand in hand with educators, as observed in the Gauteng and Free State provinces [49].

Regarding the median differences in malnutrition indicators, first we noted variations in the median HAZ, WAZ and BAZ scores. One thing that that stood out was that the values for urban school children remained stable or improved over the learning phases compared that for their rural counterparts. Learning grade is correlated with a child’s age [79] because as school children advance to intermediate learning grades, they are growing in age. Higher stunting and low mean BAZ, WAZ, and HAZ scores have been observed in rural settings versus urban settings in this study, which is comparable to the results of other African studies [13,14,15,26,30]. The prevalence of stunting [20% vs. 3%], underweight [17% vs. 2%], and thinness [28% vs. 10%] was, respectively, higher in rural than in urban school children in this study, and while the prevalence of overweight/obesity was high in both settings, it was greater in the urban setting [28% vs. 19%], similar to other studies in LMICs [16,80]. Furthermore, the prevalence of overweight/obesity in the urban setting was almost double that in the rural setting, although the prevalence were high in both settings, and this has been attributed to a shift from traditional diets to energy-dense diets (i.e., nutrition transition) [21,24], which the present findings infer—the school food and nutrition environment is not suitable to promote the consumption of nutritious foods.

Rural–urban setting malnutrition indicators showed that school children in the urban setting were less likely to be stunted, underweight and thin, but almost twice as likely to be overweight/obese compared to the rural children, as found in other reports in African and developing countries [13,14,15,26,30,41,59]. Both stunting and underweight were lower among the urban versus rural school children. Malnutrition indicators per setting showed that boys and school children in the intermediate learning phase were more likely to be stunted in the rural setting. In the urban setting, school children living in households with an income of USD 52.51 to USD 262.60/month and receiving the CSG were more likely to be thin, yet those from households with the same income of USD 52.51 to USD 262.60/month were also more likely to be overweight/obesity in the urban setting, as observed in several studies [35,36,37] and explained by the differences in the standard of living in the two settings [36]. To ensure achieving the UN’s SDG goals 1, 2, and 3, the South African government introduced the National Development Plan (NDP) vision 2023, adopted in 2012 with the objective to reduce child malnutrition through eradicating poverty in all its forms, by provision of social grants [81]. The majority of school children in the rural setting were recipients of the CSG in this study, indicating a low socio-economic status, especially when a caregiver earns less than ZAR 48,000/year [82]. Therefore, high unemployment and receiving the CSG in this study correlate with the presence of undernutrition, especially in the rural setting. This further indicates that the CSG is not meeting its objective to address malnutrition due to persistent undernutrition that is still observed among children in general. Researchers have attributed this to the misuse of the CSG by parents, prioritizing on other household expenses rather than enhancing the well-being of a child [83]. 

Lastly, although not all caregivers in the current study were the biological parents/mothers to these children, the literature documents the closeness between a mother and a child sharing resources and a similar nutritional status [55,84]. In addition to the socio-economic background, parental/maternal factors have been elucidated as the main determinants of malnutrition in children [2,4,85]. Also, the impact of the social status of fathers on their children’s nutrition and health has been acknowledge, but fathers are still underrepresented in child nutritional status research and interventions [86]. Nonetheless, the effects of parental education and employment status on child nutritional outcomes show the ability to acquire health knowledge on recommended feeding practices, as well as increased resources for food access, affordability, and availability [34,35,55]. Therefore, the social and economic situation observed—a disproportionate burden of poverty and unemployment—continues to predispose children to malnutrition. Much as some researchers [87] have alluded to, the CSG has slightly improved the poverty situation in South Africa, where over two-thirds of children are from poor backgrounds, below the poverty line [88]. Further, we cannot ignore that the COVID-19 pandemic has exacerbated poor child nutrition outcomes, destabilizing household food security. Considering the substantial effects of malnutrition on neurological development as well as the behavioral capacity of children, resulting in declining academic performance through reduced learning capacity and poor school attendance [89], these children may never reach their full scholastic potential. 

### Limitations of This Study

The cross-sectional study design and smaller sample size in rural settings versus that of urban settings restrict the causal inference and generalizability of the findings. Secondly, a social desirability bias may have been introduced during interviews; as a result, we acknowledge these reporting and recall biases, which might have had some influence on self-reported data from caregivers. Third, collecting data from child–mother pairs for this kind of investigation is ideal; however, the unavailability of mothers, especially in the GUS, due to work commitments among other reasons led to collecting data from caregivers of which several were not biological parents. Hence, we could not quantify important maternal data such as health status, obstetric history, and anthropometry. Furthermore, the involvement of fathers was not investigated in this study, so we call for better conceptualized future studies to simultaneously and comprehensively study paternal factors with maternal factors. Further robust multilevel modelling to account for rural–urban variations in comprehensive and detailed socio-demographic-associated factors should be considered in future studies as well as quantifying dietary intake and habits for school children. We must also consider the socio-cultural context in their communities as well as seasonality, which might affect nutritional status and food security. South Africa has broad cultural diversity both between provinces and even within a province; therefore, the level of ethnic diversity is also an important factor to be considered in the future. Nonetheless, this study has been able to compare malnutrition indicators and associated socio-demographic factors among children in rural Mpumalanga and urban Gauteng in South African public primary schools.

## 5. Conclusions 

This study highlights a significantly high prevalence of stunting, underweight and thinness among rural school children compared to their urban counterparts. While overweight/obesity was present in both groups, the urban children were almost twice as likely to be overweight/obese compared to the rural children. In addition to sex and the learning phase, socio-economic factors such as a household income, employment status and receiving the CSG were the largest contributors to rural–urban differences among school children associated with either of the malnutrition indicators both in bivariate and multivariate analyses. This affirms that a poor socio-economic status accounts more for malnutrition in rural–urban disparities among school children. Nutritional intervention approaches should be adjusted to socio-economic contexts to effectively tackle malnutrition. These findings may enlighten policy makers when planning initiatives for diverse regions by simplifying implementation of recommendations.

## Figures and Tables

**Table 1:** Comparison of characteristics of caregivers by setting.

Variables	Categories	All *n* (%)	Rural *n* (%)	Urban *n* (%)	Chi-Square/Fisher Exact Test *p*
Caregivers age (years)	<35	123 (28)	109 (28)	144 (28)	0.968
	≥35	650 (72)	281 (72)	369 (72)	
Marital status	Single	491 (56)	264 (68)	227 (44)	<0.0001
	Married	332 (35)	85 (22)	247 (48)	
	Divorced/Widow	80 (9)	41 (11)	39 (8)	
Education level	No schooling	40 (4)	29 (7)	11 (2)	<0.0001
	Primary	110 (13)	86 (22)	24 (5)	
	Secondary	216 (25)	118 (30)	98 (19)	
	Grade 12	222 (24)	76 (19)	146 (28)	
	Tertiary	315 (34)	81 (21)	234 (46)	
Employed	No	437 (51)	259 (66)	178 (35)	<0.0001
	Yes	466 (49)	131 (34)	335 (65)	
Receiving CSG	No	496 (50)	101 (26)	395 (74)	<0.0001
	Yes	407 (50)	289 (77)	118 (23)	
Household head	Self	520 (59)	262 (68)	258 (50)	<0.0001
	Partner/Spouse	190 (20)	43 (12)	147 (29)	
	Parents	55 (12)	24 (6)	86 (17)	
	Relatives	74 (9)	52 (14	22 (4)	
Household income (monthly)	<USD 52.51	489 (55)	259 (66)	230 (45)	<0.0001
	USD 52.51–USD 262.60	204 (23)	109 (28)	95 (19)	
	USD 262.60–USD 525.20	210 (22)	22 (6)	188 (37)	
	>USD 525.20	0 (0)	0 (0)	0 (0)	
Source of energy	Electricity	888 (98)	378 (97)	510 (99)	<0.0001
	Other	15(2)	12 (3)	3 (1)	
Refrigerator use	No	390 (36)	251 (64)	139 (8)	<0.0001
	Yes	513 (64)	43 (36)	470 (92)	
Access to water	No	20 (3)	20 (5)	2 (0)	<0.0001
	Yes	880 (97)	370 (95)	510 (100)	

**Table 2:** Comparison of characteristics of children and their malnutrition indicators by setting.

Variables	Categories	All *n* (%)	Rural *n* (%)	Urban *n* (%)	Chi-Square/Fisher Exact Test *p*
Age (years)	6–9	460 (58)	283 (73)	222 (43)	<0.0001
	≥10	398 (42)	107 (27)	291 (56)	
Child sex	Boys	441 (50)	206 (53)	235 (46)	<0.0001
	Girls	390 (50)	184 (47)	206 (54)	
Learning phases	Foundation	526 (61)	302 (77)	224 (44)	<0.0001
	Intermediate	297 (39)	88 (23)	289 (56)	
Underweight	Yes	45 (10)	40 (17)	5 (2)	<0.0001
Stunting	Yes	93 (12)	77 (20)	16 (3)	<0.0001
Thinness	Yes	116 (18)	95 (28)	21 (7)	<0.0001
Overweight/obesity	Yes	194 (24)	55 (19)	139 (28)	<0.0001

**Table 3:** Bivariate associations of malnutrition indicators with caregiver’ socio-demography.

	Rural			Urban		
Variables Categories	Stunting *p* *n* (%)	Thinness *p* *n* (%)	Ov/Ob *p* *n* (%)	Stunting *p* *n* (%)	Thinness *p* *n* (%)	Ov/Ob *p* *n* (%)
Marital status (*p*)	0.995	0.539	0.959	0.347	0.856	0.706
Single	52 (20)	67 (30	37 (19)	6 (3)	10 (6)	65 (30)
Married	17 (20)	17 (23)	12 (18)	10 (4)	9 (5)	65 (27)
Divorced/Widow	8 (21)	11 (31)	6 (20)	0 (0)	2 (7)	9 (24)
Education level (*p*)	0.320	0.824	0.989	0.476	0.857	0.989
No schooling	8 (28)	8 (31)	3 (14)	1 (9)	1 (13)	3 (30)
Primary	14 (17)	18 (19)	13 (19)	1 (4)	1 (6)	23 (30)
Secondary	18 (16)	27 (27)	17 (19)	5 (5)	5 (7)	93 (30)
Grade 12	19 (25)	22 (33)	10 (19)	3 (2)	6 (5)	39 (28)
Tertiary	18 (23)	20 (29)	12 (20)	6 (3)	8 (5)	62 (27)
Employed (*p*)	0.815	0.249	0.849	0.444	0.969	0.020
No	52 (20)	68 (30)	35 (18)	7 (4)	8 (6)	37 (22)
Yes	25 (19)	27 (24)	20 (19)	9 (3)	13 (6)	102 (32)
Receive CSG (*p*)	0.134	0.203	0.365	0.161	0.029	0.961
No	5 (25)	9 (23)	18 (22)	10 (3)	12 (4)	108 (28)
Yes	52 (18)	76 (30)	37 (17)	6 (5)	9 (10)	31 (28)
H. income/month (*p*)	0.158	0.034	0.542	0.576	0.122	0.186
<USD 52.51	52 (20)	68 (30)	35 (18)	9 (4)	(5)	54 (24)
USD 52.51–USD 262.60	24 (22)	27 (28)	14 (17)	3 (3)	7 (11)	30 (34)
USD 262.60–USD 525.20	1 (5)	0 (0.0)	6 (27)	4 (2)	5 (4)	55 (30)

CSG stands for the child social grant, H. income stands for household income, Ov/Ob stands for overweight/obesity, and *p* stands for the *p*-value.

**Table 4:** The association of settings with malnutrition indicators.

Setting	OR (95% CI)	*p*	AOR (95% CI)	*p*
Stunting				
No	1		1	
Yes	0.13 (0.07–0.23)	≤0.0001	0.33 (0.13–0.87)	0.024
Underweight				
No	1		1	
Yes	0.15 (0.09–0.25)	≤0.0001	0.16 (0.06–0.42)	≤0.0001
Thinness				
No	1			
Yes	0.11 (0.04–0.29)	≤0.0001	(collinearity)	
Overweight/obesity				
No	1			
Yes	1.72 (1.21–2.44)	0.003	(Collinearity)	

**Table 5:** The association of stunting with covariates in the rural setting.

Malnutrition Indicator	OR (95% CI)	*p*	AOR (95% CI)	*p*
Stunting				
Sex				
Girls	1		1	
Boys	0.41 (0.25–0.69)	0.001	0.53 (0.30–0.94)	0.029
Learning grades				
Foundation phase	1		1	
Intermediate phase	8.63 (4.95–1506)	≤0.0001	7.87 (4.48–13.82)	≤0.0001

**Table 6:** The association of thinness and overweight/obesity with covariates in the urban setting.

Malnutrition Indicator	OR (95% CI)	*p*	AOR (95% CI)	*p*
Thinness				
CSG				
No	1		1	
Yes	2.64 (1.07–6.50)	0.034	2.49 (0.90–6.86)	0.078
Household income/month				
<USD 52.51	1		1	
USD 52.51–USD 262.60	2.79 (0.99–7.88)	0.052	2.89 (1.01–8.24)	0.047
USD 262.60–USD 525.20	0.76 (0.25–2.33)	0.636	1.18 (0.32–3.91	
Overweight/Obesity				
Sex				
Girls	1		1	
Boys	0.80 (0.54–1.19)	0.264	0.81 (0.54–1.20)	0.289
Household income/month				
<USD 52.51	1		1	
USD 52.51–USD 262.60	1.80 (1.03–3.12)	0.038	1.80 (1.02–3.10)	0.042
USD 262.60–USD 525.20	1.35 (0.87–2.09)	0.177	1.35 (0.87–2.09)	0.177

## Data Availability

The dataset for the study group generated and analyzed during the current study is available from the corresponding author upon reasonable request.
